# Altered glycosylation in pancreatic cancer and beyond

**DOI:** 10.1084/jem.20211505

**Published:** 2022-05-06

**Authors:** Jan C. Lumibao, Jacob R. Tremblay, Jasper Hsu, Dannielle D. Engle

**Affiliations:** 1 Salk Institute for Biological Studies, La Jolla, CA

## Abstract

Pancreatic ductal adenocarcinoma (PDA) is one of the deadliest cancers and is projected to soon be the second leading cause of cancer death. Median survival of PDA patients is 6–10 mo, with the majority of diagnoses occurring at later, metastatic stages that are refractory to treatment and accompanied by worsening prognoses. Glycosylation is one of the most common types of post-translational modifications. The complex landscape of glycosylation produces an extensive repertoire of glycan moieties, glycoproteins, and glycolipids, thus adding a dynamic and tunable level of intra- and intercellular signaling regulation. Aberrant glycosylation is a feature of cancer progression and influences a broad range of signaling pathways to promote disease onset and progression. However, despite being so common, the functional consequences of altered glycosylation and their potential as therapeutic targets remain poorly understood and vastly understudied in the context of PDA. In this review, the functionality of glycans as they contribute to hallmarks of PDA are highlighted as active regulators of disease onset, tumor progression, metastatic capability, therapeutic resistance, and remodeling of the tumor immune microenvironment. A deeper understanding of the functional consequences of altered glycosylation will facilitate future hypothesis-driven studies and identify novel therapeutic strategies in PDA.

## Teaser

Glycosylation alterations are a hallmark of pancreatic cancer. This review discusses the contributions of aberrant glycosylation to protumorigenic signaling, metastasis, and remodeling the tumor immune microenvironment in pancreatic cancer.

## Introduction

For the past 40 yr, survival rates for patients diagnosed with pancreatic ductal adenocarcinoma (PDA) have remained at <10% (Abbassi and Algül, 2019). Due to increasing incidence, difficulty in early diagnosis, and the refractory nature of advanced disease, PDA is projected to become the second leading cause of cancer death by 2040 (Abbassi and Algül, 2019). For years, gemcitabine has been the standard of care for PDA patients, providing median survival times of ∼6 mo (Abbassi and Algül, 2019). Although subsequent clinical trials demonstrated improved survival benefits of FOLFIRINOX (11.1 mo) and gemcitabine/nab-paclitaxel (8.5 mo), systemic chemotherapeutic regimens are rarely curative in PDA. Currently, surgical resection remains the only curative option, yet most patients are ineligible due to regional or distal metastases at the time of diagnosis (Kleeff et al., 2016).

In a concerted effort to provide more robust survival benefits for PDA patients, significant research strides in recent years have contributed substantially to our understanding of PDA. These studies have identified key driver mutations, signaling pathways, and tumor-microenvironment interactions that promote disease progression, regulate metastasis, and drive therapeutic resistance. Yet, the impact of glycosylation, one of the most common post-translational modifications which compounds the complexity of intra- and intercellular regulation, remains understudied in PDA. The paucity of such research in this area is driven by the vast aggregate complexity of glycans and their associated structures, the intricate mechanisms by which glycosylation and glycan assembly are regulated, and the lack of models that recapitulate glycosylation in human disease. For instance, only in recent years has a mouse model of pancreatic disease capable of producing the PDA-associated glycan CA19-9 been developed (Engle et al., 2019), providing evidence for the functional capacity of a glycan that was heretofore only considered a correlative biomarker of disease progression. Understanding the functional roles of glycosylation in PDA will be crucial for identifying both new diagnostic tools and therapeutic targets.

## Landscape of glycosylation

Glycans exist as covalent linkages of saccharides in either free form or, more commonly, attached to proteins or lipids as glycoconjugates, thus forming glycoproteins, glycolipids, and proteoglycans (Ohtsubo and Marth, 2006; Fuster and Esko, 2005). In mammals, glycans are constructed from a combination of 10 monosaccharides (galactose, glucose, mannose, fucose, xylose, *N*-acetyl-galactosamine, *N*-acetyl-glucosamine, glucuronic acid, iduronic acid, and sialic acid), which are attached via α- or β-glycosidic bonds to form linear or branched structures (Kudelka et al., 2015). Glycan chain assembly is catalyzed by glycosyltransferases and glycosidases, which are encoded by 1–2% of the human genome (Taniguchi and Kizuka, 2015). Biochemically, glycans can be classified into families. *N*-glycans are linked via an amide bond to asparagine in the consensus Asn-X-Ser/Thr sequon (where X is any amino acid except proline). Structurally, *N*-glycans present as branched oligomannose, complex, or hybrid moieties. *O*-glycans are most commonly linked to the oxygen atom on serine or threonine, and in some cases tyrosine. These can be further subdivided into *O*-linked GlcNAc, as well as secreted or membrane glycoproteins decorated with *O*-glycans, the most common of which is the mucin-type (or GalNAc type) *O*-glycan. In contrast to *N*-glycans, no glycosite amino acid sequon has been identified for *O*-GalNAc-linked glycans (Kudelka et al., 2015).

Other glycan families include free or proteoglycan-conjugated long linear repeats of disaccharide units called glycosaminoglycans, ceramide-linked glycans called glycosphingolipids, and glycosylphosphatidylinositol-linked proteins (Fuster and Esko, 2005). Individual saccharides in glycan chains can be further modified, e.g., by phosphorylation, sulfation, and acetylation, increasing their structural diversity. Glycans are often found as secreted glycoconjugates or on the cell surface, forming a multifunctional layer of glycans termed the glycocalyx. The glycocalyx is responsible for a variety of biological functions including maintaining vascular permeability (Uchimido et al., 2019), immune cell recognition (Möckl, 2020), and crosstalk with the extracellular matrix (ECM; Kang et al., 2018).

In contrast to proteins, the sequence of glycan chains is not genetically encoded. Instead, glycan assembly and the formation of glycoconjugates are regulated by numerous factors, including the availability of nucleotide sugars that act as donor substrates (e.g., UDP-galactose, UDP-*N*-acetylglucosamine, GDP-fucose, and CMP-*N*-acetylneuraminic acid), acceptor substrates, and cofactors, as well as the glycosyltransferases and glycosidases necessary to catalyze such reactions. Further, each of these elements must be localized appropriately within the cell and secretory apparatus. This process is governed by molecular chaperones, endogenous lectins, and nucleotide sugar transporters (Taniguchi and Kizuka, 2015). The collection of enzymes that comprise the glycosylation machinery is primarily localized to the Golgi, with some also present in the endoplasmic reticulum (Taniguchi and Kizuka, 2015).

The vast diversity of the glycome, and the predominant presence of glycans on membranes and secreted proteins, positions glycoconjugates as mediators of multiple biological processes such as inter- and intracellular signaling, cell adhesion and motility, and immune regulation. Investigation of the functional roles of aberrant glycans has been difficult not only in part due to their complexity and families of glycosyltransferases with redundant functions, but also because of differences in the glycome between mouse models and humans. For example, mice endogenously display glycan epitopes not normally observed in humans, including α-Gal and Neu5Gc epitopes (Saleh et al., 2020; Altman and Gagneux, 2019). Conversely, sLe^a^ (CA19-9) is frequently upregulated in human pancreatic disease, but its synthesis is precluded in mice due to *Fut3*, the only enzyme capable of transferring the core α-1,4-fucose required for sLe^a^ synthesis, being a pseudogene in mice (Engle et al., 2019). Understanding glycosylation alterations, the regulatory aberrations underpinning them, and their consequences in the progression of malignant disease provide a promising and critical avenue to identify potential therapeutic vulnerabilities and deepen our understanding of cancer.

## Aberrant glycosylation contributes to pancreatic cancer phenotypes

Evidence of altered glycosylation in cancer emerged in the 1970s, when glycopeptides isolated from transformed cell lines were observed to be larger than those from non-transformed cells (Buck et al., 1971). Around the same time, it was realized that many of the oncofetal and tumor-associated antigens used to detect and track disease progression were in fact carbohydrates (Feizi, 1985). Since then, substantial profiling of glycosylation alterations in various cancers has reshaped our understanding of how extensively glycosylation changes during the onset and progression of malignant disease (Munkley and Elliott, 2016; Gupta et al., 2020). Some of the most prevalent glycosylation irregularities described in PDA include dysregulated *O*-GlcNAc, increased sialylation and fucosylation, aberrantly branched *O*-glycan structures, and altered mucins (Qorri et al., 2020). In turn, these alterations impact various pro-tumorigenic signaling pathways, promote metastatic phenotypes, and remodel the tumor immune microenvironment. In the following sections, we describe the contributions of altered glycosylation in PDA to these phenotypes and highlight areas in which therapeutic vulnerabilities may exist.

## Sustaining proliferative and pro-tumorigenic signaling

### Intracellular signaling events regulated by glycosylation

**Figure 1.**
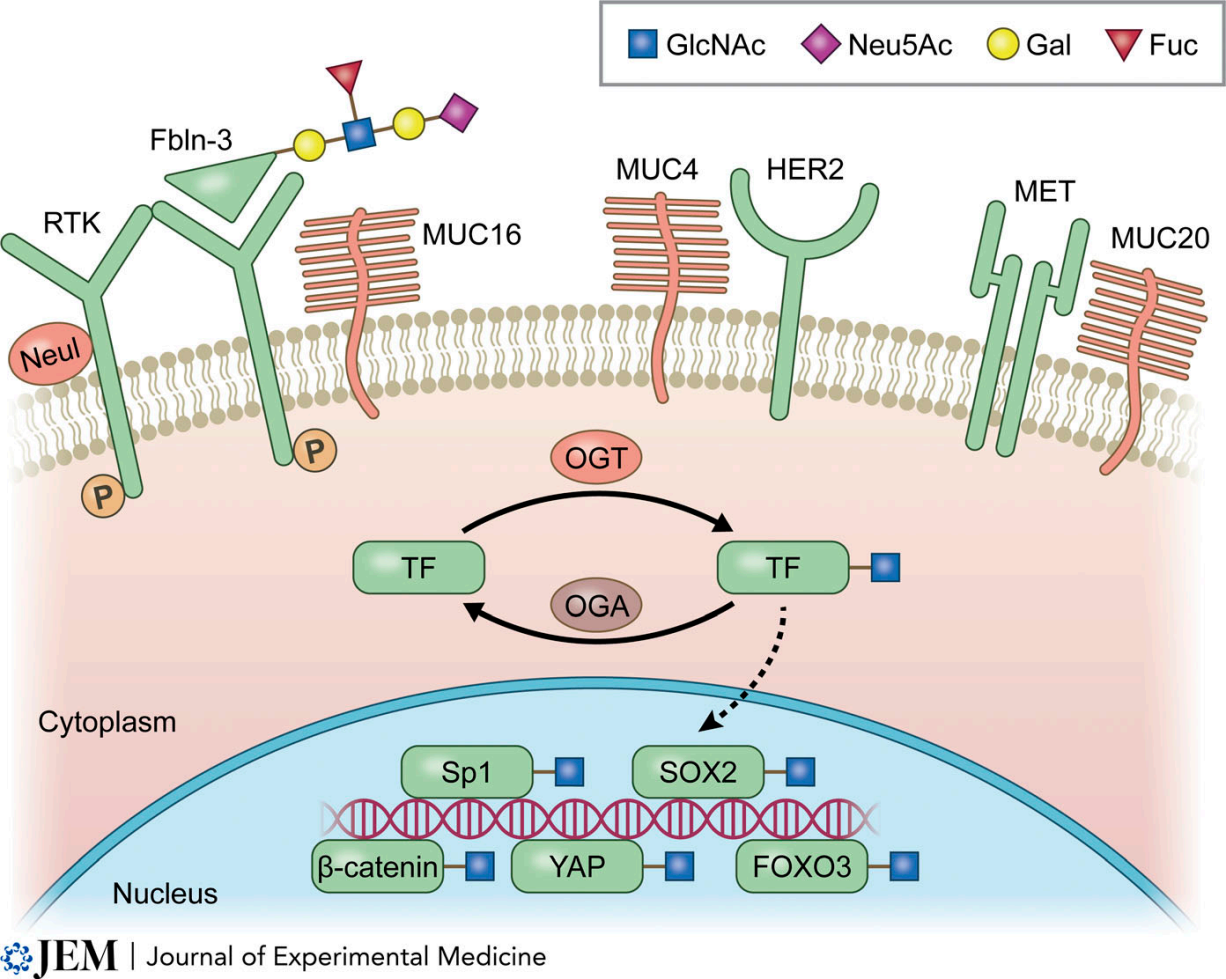
**The impact of aberrant glycosylation on cell signaling in PDA.** *O*-GlcNAcylation of transcription factors catalyzed by OGT, such as Sp1, SOX2, β-catenin, YAP, and FOXO3, has been demonstrated to regulate their nuclear localization and activity. At the cell membrane, associations of mucins such as MUC16, MUC4, and MUC20 with RTKs such as EGFR, HER2, and MET have been described to modulate downstream signaling. Enzymatic activity of Neu1 on cell surface receptors has been further shown to impact receptor dimerization. Modification of receptor ligands such as Fibulin-3 (Fbln3) has also been demonstrated to increase affinity for EGFR.

Glycosylation-mediated intracellular signaling is primarily mediated by the *O*-linked β-*N*-acetylglucosamine (*O*-GlcNAc) modification. Levels of *O*-GlcNAc are increased in breast, colon, and pancreatic cancer (Nagel and Ball, 2015). *O*-GlcNAc modification alters the localization and activity of metabolic enzymes, histones, and transcriptional regulators (Fig. 1; Nagel and Ball, 2015). *O*-GlcNAc modification of transcription factors, including Sp1, β-catenin, SOX2, FOXO3, and YAP, regulates their nuclear translocation and activity, subsequently promoting gene expression programs that confer proliferative and anti-apoptotic cancer cell phenotypes (Banerjee et al., 2013; Jia et al., 2020; Sharma et al., 2019; Shin et al., 2018; Peng et al., 2017). Levels of *O*-GlcNAc are governed by the balance between *O*-GlcNAc transferase (OGT) and *O*-GlcNAcase (OGA), which add and remove UDP-GlcNAc to and from acceptor substrates, respectively. In addition to OGT and OGA, *O*-GlcNAcylation is sensitive to the nutritional and metabolic status of cells. The dense fibrotic and hypovascular nature of PDA leads to a hypoxic niche, increasing flux through glycolysis and glutamine metabolism (Guillaumond et al., 2014), pathways that converge toward the hexosamine biosynthetic pathway (HBP). The HBP is a branch of glycolysis responsible for the production of UDP-GlcNAc, a key substrate for glycosylation. HBP activity is increased in PDA and associated with poor survival (Jia et al., 2020). O-GlcNAcylation, hypoxia, and HPB converge in PDA. Hypoxia increases levels of *OGT*, *OGA*, and *O*-GlcNAc, and increases expression of glutamine–fructose-6-phosphate transaminase 1 (*GFPT1*), which catalyzes the first and the rate-limiting step of the HBP (Guillaumond et al., 2013). Pharmacologic targeting of the HBP using antagonists of GFPT disrupts *O*-GlcNAc protein modification, decreases cell proliferation, survival, and invasion, and increased starvation-induced apoptosis (Guillaumond et al., 2013; Jia et al., 2020). Thus, the centrality of the HBP in fueling aberrant protein glycosylation in cancer presents a promising therapeutic vulnerability.

In addition to *O*-GlcNAc, other types of glycosylation also impact intracellular signaling. Increased *O*-GalNAcylation, catalyzed by GalNAc transferases that are upregulated in PDA (Taniuchi et al., 2011; Sutherlin et al., 1997), renders malignant cells resistant to apoptosis by regulating the processing of caspase-3, -8, -9, and Bid. In addition to *O*-glycosylation, modification and stabilization of other intracellular proteins with sialic acid have been described, such as sialylation of HIF-1α (Jones et al., 2018).

Collectively, these studies highlight a dynamic interplay between the remodeled PDA tumor microenvironment and metabolically and genetically reprogrammed tumor cells that influence glycosylation-mediated intracellular signaling events and promote proliferative and anti-apoptotic phenotypes. By targeting the metabolic dependencies controlling these processes, pro-tumorigenic intracellular signaling mediated by aberrant *O*-GlcNAcylation can be abrogated.

### Glycosylation mediated pro-tumorigenic cell surface receptor signaling

Like intracellular proteins, cell membrane receptor activity is also tunable via glycosylation modifications. Levels of the enzyme ST6Gal-I, which gives rise to α-2,6-sialylation, are increased in acinar-to-ductal metaplasia, pancreatitis, preinvasive pancreatic intraepithelial neoplasms, and PDA (Schultz et al., 2016). Increased α-2,6-sialylation of TNFR1 inhibits internalization and stabilizes signaling through AKT and NF-κB, conferring resistance to gemcitabine and TNF-induced apoptosis (Britain et al., 2017; Holdbrooks et al., 2018; Schultz et al., 2016; Chakraborty et al., 2018). Dimerization of several receptor tyrosine kinases and toll-like receptors are regulated by the sialidase neuraminidase 1 (Neu1), which cleaves terminal sialic acid residues from glycoproteins (Abdulkhalek and Szewczuk, 2013; Jayanth et al., 2010; Fig. 1). Specifically, ligand binding induces a receptor conformational change that results in MMP9 and Neu1 activation. This leads to the hydrolysis of terminal α-2,3-sialyl residues present on receptors, thereby removing steric hindrance and allowing RTK dimerization (Gilmour et al., 2013; Mathew et al., 2015; Mathew et al., 2016). Previous reports in lung cancer cells demonstrated that increased *N*-sialylation or *N*-fucosylation of EGFR suppresses dimerization and attenuates the activation of downstream signaling (Liu et al., 2011). Notably, EGFR activation is required for KRAS-induced pancreatic tumorigenesis (Ardito et al., 2012), underscoring a critical role for glycosylation in regulating epithelial transformation. Neu1-dependent RTK activation represents a targetable vulnerability, with aspirin and neuraminidase inhibitors such as Tamiflu capable of inhibiting Neu1 activity and preventing EGFR phosphorylation and downstream signaling (Qorri et al., 2020). Additionally, changes in *N*-glycosylation branching have been shown to increase metastatic dissemination of multiple cancer types and are correlated with PDA progression (Pan et al., 2014). In PDA, proteins with altered *N*-glycosylation sites or structures often play significant roles in pathways that increase metastasis such as the TGF-β and NF-κB signaling pathways (Pan et al., 2012).

Stabilization of cell membrane receptors is also crucial to their activity, and it is influenced by high molecular weight glycoproteins called mucins, which function canonically in lubrication, cell signaling, and barrier formation. Mucins have been reviewed extensively elsewhere (Suh et al., 2017). In PDA, the global expression of mucins is altered (Yamada et al., 2006). Mucins potentiate pro-tumorigenic signaling via a variety of mechanisms (Fig. 1). For instance, MUC16 interacts with EGF receptors to activate AKT and GSK3β signaling, an effect that can be reversed using anti-MUC16 antibodies (Thomas et al., 2021). Similarly, MUC4 interacts with and stabilizes HER2, and MUC4 knockdown in CD14/HPAF and Capan1 cells lead to reduced phosphorylation of the downstream effectors FAK and ERK (Chaturvedi et al., 2008). MUC20 interacts with MET, and knockdown of MUC20 in HPAC and HPAF-II cells decreased HGF-mediated phosphorylation of MET (Chen et al., 2018). Engagement of these mucin-mediated cell signaling programs results in pro-tumorigenic changes in cell behavior, including increases in cell viability, pancreatic stellate cell-induced migration and invasion, and in vivo orthotopic tumor growth (Chen et al., 2018). The resulting impact of mucins on patient outcomes is also clear. The expression of MUC4, MUC16, and MUC20 are all correlated with poor survival (Jonckheere and Van Seuningen, 2018; Ohya et al., 2017; Remmers et al., 2013; Haridas et al., 2011; Nagata et al., 2007; Ringel and Löhr, 2003; Yonezawa et al., 2002; Yonezawa and Sato, 1997). Notably, dense *O*-glycosylated mucins contribute to glycocalyx formation and can hinder the uptake of chemotherapies such as 5-fluorouracil, an effect that can be reversed by global inhibition of *O*-glycosylation with benzyl-α-GalNAc (Kalra and Campbell, 2009; Kalra and Campbell, 2007).

In addition to modifying receptors, the glycan decoration of ligands serves as another mechanism by which cell surface signaling is altered in the pancreatic disease. sLe^a^/CA19-9 directly impacts the activation of EGFR and potentiates downstream signaling in pancreatic disease by decorating the EGFR ligand Fibulin-3 (Engle et al., 2019; Fig. 1). Mice genetically engineered to produce CA19-9 develop severe pancreatitis. In the KRAS-mutant context, CA19-9 elevation dramatically decreased survival (Engle et al., 2019). Importantly, pancreatitis severity and EGFR hyperactivation were attenuated by the treatment of mice with anti–CA19-9 blocking antibodies, including the fully human 5B1 clone (Weitzenfeld et al., 2019), highlighting a glycosylation-based therapeutic vulnerability in pancreatitis and PDA. 5B1 is currently being evaluated as an imaging modality and a therapeutic agent in clinical trials (Houghton et al., 2017; Lohrmann et al., 2019).

## Role of glycosylation in PDA invasion and metastasis

### Cell surface sialylation and fucosylation-mediated adhesion and invasion

**Figure 2.**
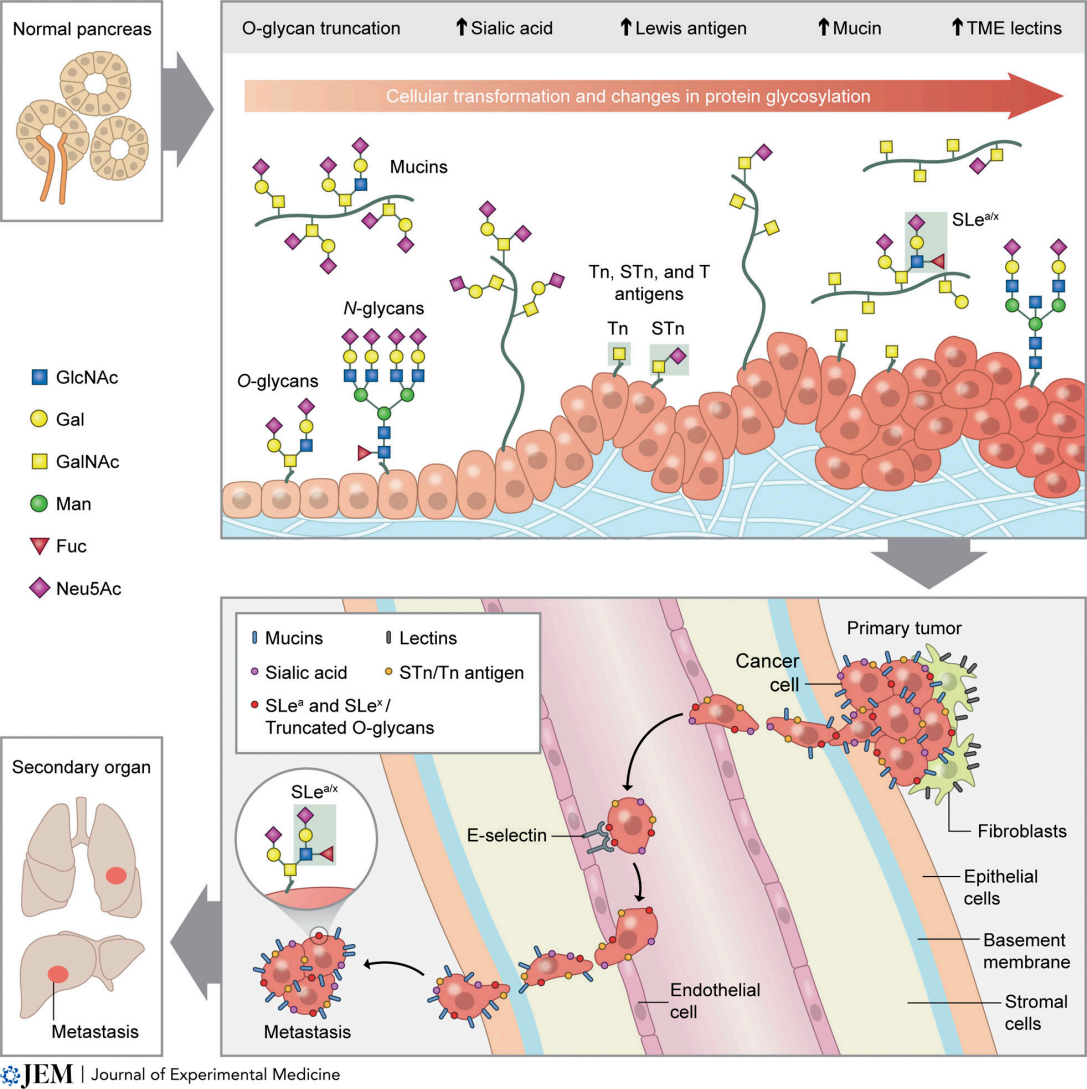
**The impact of aberrant glycosylation alterations on cancer progression, cell adhesion, and metastasis.** Disease progression in the pancreas correlates with alterations in core *O*- and *N*-glycan moieties. These include truncated *O*-glycans, increased sialylation, mucin deposition, Lewis blood group antigen decoration, and tumor microenvironment (TME) lectin expression. These alterations in protein and lipid glycosylation can drive changes in cell adhesion and metastasis, enabling more efficient colonization of distant organs such as the lung and liver. The secondary metastatic sites often recapitulate the altered glycosylation landscape of the primary tumor.

Early investigations into how glycosylation changes influence metastasis focused on the sialylated and fucosylated Lewis blood group antigens that include sialyl-Lewis x (sLe^x^) and sialyl-Lewis a (sLe^a^, CA19-9). Upregulation of these glycan moieties in tissue and serum of PDA patients was discovered in the 1980s (Haglund et al., 1986; Kalthoff et al., 1986; Pour et al., 1988; Singh et al., 2015; Tang et al., 2015; Balmaña et al., 2015). Several subsequent ground-breaking publications demonstrated the potential of these antigens to increase the binding of circulating tumor cells to E-selectin on endothelial cells, potentially facilitating metastasis (Fig. 2; Phillips et al., 1990; Iwai et al., 1993; Kaji et al., 1995; Takada et al., 1991; Takada et al., 1993; Takada et al., 1995; Sawada et al., 1994). Conversely, engineering cells to divert glycosylation away from sLe^a^ production reduced E-selectin adhesion and metastases in transplanted mice (Aubert et al., 2000). Following metastatic progression, cell surface sialylation is also increased, with metastatic nodules displaying higher sialylated-GalNAc (sTn, a common antigen in cancer and the subject of several cancer vaccine studies) levels than the primary tumor or normal tissue (Itzkowitz et al., 1991; Fig. 2). Glycoengineering to increase metabolic flux through the sialic acid production pathway increases cell surface sialic acid concentration and consequently increases cellular binding to E- and L-selectin (Almaraz et al., 2012; Mathew et al., 2016), a key step in circulating tumor cell extravasation into distant tissues. Conversely, neuraminidase treatment to remove sialic acid on human PDA cells causes decreased adhesion to ECM (Sawada et al., 1993). Additionally, increases in tumor cell surface sialylation led to increases in the adhesive and invasive properties of tumor cells. Elevated expression of fucosyltransferases and sialyltransferases, such as FUT3, -5, -6, ST6Gal1, and ST3Gal3/4, similarly led to increases in cell motility and metastatic potential in both cell lines and mouse models (Gao et al., 2019; Bassagañas et al., 2014a; Bassagañas et al., 2014b; Pérez-Garay et al., 2013; Pérez-Garay et al., 2010; Hsieh et al., 2017; Britain et al., 2021).

In addition to sialyl Lewis antigens and changes in cell surface sialylation and fucosylation, alterations of other glycoproteins and glycan moieties, such as mucins and truncated *O*-glycan chains (Tn, sTn), potentiate PDA metastasis (Fig. 2; Gendler, 2001; Kohlgraf et al., 2003; Satoh et al., 2000; Hanson et al., 2016; Swanson et al., 2007; Mcdermott et al., 2001; Singh et al., 2007). In PDA, MUC1 interacts with both E- and P-selectins (Mcdermott et al., 2001) to facilitate extravasation and transmits signals into the cell from the surrounding microenvironment to induce increased invasion through stabilization and phosphorylation of FRA-1 and c-JUN (Hanson et al., 2016; Besmer et al., 2011). Similar results have also been reported for MUC16 in human cell lines (Muniyan et al., 2016). Meanwhile, the elevation of truncated *O*-glycan chains such as Tn and sTn occurs because of aberrations in *O*-glycosylation extension enzymes such as core synthase 3 and C1GALT1, resulting in enhanced cellular invasion, migration, and metastasis (Radhakrishnan et al., 2014; Hofmann et al., 2015; Chugh et al., 2018; Kuo et al., 2021b).

Collectively, these studies support a causal role for Lewis antigens, sialylation, fucosylation, mucins, and Tn/sTn in enhancing metastatic capability via interactions with ECM and glycan-binding lectins on the endothelium. However, the nuanced impacts of different sialyltransferases on PDA behavior underscore the complexity of glycosylation events and the need for careful consideration of how changes in glycosyltransferases affect the global glycome and cell function (Holst et al., 2017). Characterization of discrete glycosyltransferase and glycoconjugate contributions to different stages of the metastatic cascade will prove key to deepening our understanding of how to combat malignant spread.

### Altered glycosylation in the extracellular space influences cell adhesion and metastasis

Glycoconjugates often impact cellular motility, adhesion, and invasive capacity through their action in the extracellular space. For example, glycosaminoglycans (GAGs) are long polysaccharide chains of repeating two-sugar subunits that can have profound impacts on tumor interstitial pressure. The high anionic charge of GAGs alters the water content, and thus pressure, of tissues and tumors (Vedadghavami et al., 2020), which has implications for drug delivery (Provenzano and Hingorani, 2013). Heparan sulfate GAGs (HSGAGs), such as Syndecans (SDC) and glypicans, are bound to a proteoglycan core attached to the cell membrane and are involved in tumor development, cell adhesion, and metastasis (Nagarajan et al., 2018). SDC are often upregulated in gastric and colon cancers, but Syndecan-1 (SDC1) is upregulated only in pancreatic cancer, where it correlates with invasion of cancer and a poor prognosis (Conejo et al., 2000; Yao et al., 2019). Increased levels of heparanase (HPA) in pancreatic cancer increase metastatic potential by facilitating shedding of SDC1 as well as activation of the PI3K/AKT pathway and the epithelial-to-mesenchymal transition (Yang et al., 2007; Chen et al., 2020). Both SDC1 and SDC2 cooperate with KRAS mutation to promote PDA metastasis through activation of SRC kinase and phosphorylation of ERK (Yao et al., 2019; De Oliveira et al., 2012). Membrane-anchored glypicans modulate chemokine and growth factor signaling (Kleeff et al., 1998). Glypican-1 increases PDA invasion and metastasis through the modulation of FGF2 signaling (Aikawa et al., 2008). Glypican-4 has also been implicated in PDA metastasis and recurrence by increasing stemness markers through the WNT/β-catenin pathway within tumors (Cao et al., 2018). The basement membrane HSGAG Perlecan has also been shown to be upregulated in pancreatic cancer and has a direct effect on cancer-associated fibroblast populations. The depletion of Perlecan abrogated the CAF-induced pro-metastatic niche in mice, resulting in increased survival (Vennin et al., 2019).

Unlike membrane-tethered HSGAGs, hyaluronan (hyaluronic acid or HA) is a secreted GAG that is not bound to the cell surface. The effects of HA on migration, invasion, and metastasis have been extensively reviewed elsewhere (Sato et al., 2016). HA binds to its cell surface membrane receptor CD44, which has many isoforms and splice variants, some of which are only expressed in cancer (Sato et al., 2016). HA binding of CD44 results in increased PI3K signaling, invasion, and metastasis (Teranishi et al., 2009). In PDA, HA contributes to increased interstitial fluid pressure, which collapses vasculature and creates a barrier to treatment (Provenzano et al., 2012). Enzymatic ablation of HA decreases tumor and metastatic burden in KPC mice (Jacobetz et al., 2013). In light of the significant abundance and potential contribution to pancreatic cancer of HA, a clinical trial was initiated to determine whether the disruption of HA could improve patient outcomes. However, therapeutic targeting of HA in combination with nab-paclitaxel/gemcitabine was insufficient to improve patient outcomes in a Phase II clinical trial (Hingorani et al., 2018). The complex interplay between altered glycoconjugates and the tumor microenvironment will likely necessitate combinatorial approaches to target key prosurvival networks in PDA.

The compendium of both free and membrane-bound proteoglycans, glycosaminoglycans, glycolipids, and glycosylated proteins comprises the glycocalyx, which presents as a mesh that covers the cell and is at the interface of cell–cell and cell–matrix signaling. The glycocalyx plays key functional roles in cell morphology, regulation of membrane protein diffusion, and immune cell regulation (Möckl, 2020). Alterations in the glycocalyx are capable of contributing to tumor progression by modulating cell membrane protein turnover, membrane topology, and cell morphology to facilitate biophysical interactions with the surrounding microenvironment (Al-Aghbar et al., 2022; Cruz-Chu et al., 2014; Möckl, 2020). Changes in the glycocalyx are known to be important in various types of cancers, yet there is still more to learn regarding its role in pancreatic cancer. Recently, doxycycline-inducible *KRAS* mutant cell lines were utilized to visualize the glycocalyx of PDA cells, demonstrating a mutant KRAS-dependent increase in glycocalyx size, potentially decreasing the adhesion of cells to the surrounding stroma or matrix (Möckl, 2020). The glycocalyx also has physical effects on integrins in normal and tumor contexts by changing pH or physical restriction of integrins to a specific region. While this phenomenon requires further study in pancreatic cancer, it is known that integrins are aberrantly glycosylated in cancer and are critical signaling integration hubs through which cell interactions with the matrix are transmitted. Several studies have shown that in PDA, altered glycosylation of integrins changes their adhesion properties. These integrins are no longer able to bind as effectively to the matrix or E-cadherin, thereby increasing the invasive potential of tumor cells (Kuo et al., 2021a; Bassaganas et al., 2014).

## Glycoconjugates in the PDA immune microenvironment

### Aberrant glycosylation facilitates tumor immune escape

**Figure 3.**
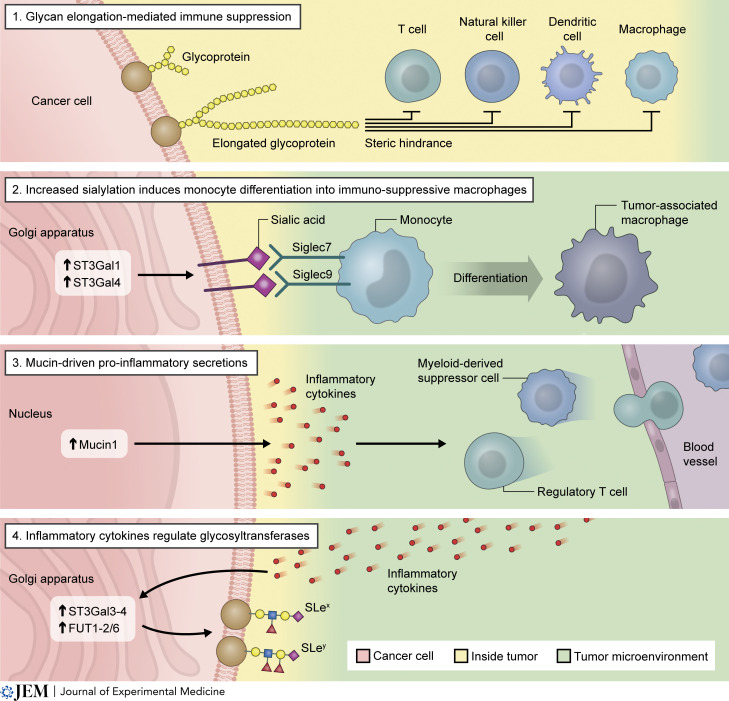
**The impact of glycosylation on the PDA immune microenvironment.** The steric hindrance from aberrant glycan elongation on the cell surface of PDA cells prevents interaction with anti-tumorigenic immune cell types, providing one mechanism of tumor cell immune escape (1). Increased expression of sialyltransferases such as ST3Gal1/4 leads to increases in tumor cell surface sialic acid that act as ligands for Siglec7/9, promoting monocyte differentiation into tumor-associated macrophages (2). Expression of MUC1 has been demonstrated to promote the release of inflammatory cytokines that play roles in regulatory T cell and myeloid-derived suppressor cell chemotaxis (3). Conversely, inflammatory cytokine signals from the surrounding microenvironment influence tumor cell glycosyltransferase expression that subsequently alters glycosylation landscapes, including expression of sialylated epitopes (4).

Glycans in cancer have long been correlated with suppressed anti-tumor immune responses (Varki et al., 2015). Glycans and glycoproteins often lie at the cell–cell interface and frequently comprise pathogen- and damage-associated molecular patterns which activate the innate immune system. By altering the cell surface glycan “code,” transformed cells can evade immune detection. Indeed, increased expression of ST3Gal1 and -4 leads to increased sialylation on PDA cells that induces monocyte differentiation into immunosuppressive tumor-associated macrophages via myeloid receptors Siglec-7 and -9 (Rodriguez et al., 2021; Fig. 3). Reciprocally, inflammatory cytokines present in the microenvironmental milieu, such as IL1β, IL6, and TNFα, upregulate PDA cell expression of ST3GAL3-4, FUT1-2, and FUT6, resulting in increased sLe^x^, SLe^y^, and α2,6-sialic acid levels, suggesting that glycosylation of PDA cells is modulated by inflammatory microenvironments (Bassagañas et al., 2015; Fig. 3).

MUC1 is overexpressed in colon, breast, ovarian, lung, and pancreatic cancers (Gendler, 2001; Sharma and Allison, 2015; Hanson and Hollingsworth, 2016; Apostolopoulos and McKenzie, 2017). Expression of MUC1 results in elevated expression of multiple proinflammatory cytokines and concomitant increases in regulatory T cell and myeloid suppressor cell populations in pancreatic tumors (Tinder et al., 2008; Fig. 3). Furthermore, cancer cells that express MUC1 display increased sialyl-Tn antigen (Dalziel et al., 2001; Gill et al., 2011; Reis et al., 1998), and alterations in MUC1 affect immune detection and increase macrophage activation through binding to sialylated MUC1, promoting tumor growth (Saeland et al., 2007; Nath et al., 1999; Cascio and Finn, 2016). Exposed cryptic peptide epitopes, as a result of truncated glycosylation, can be recognized by antibodies and are ideal targets for therapy. The potential utility of various MUC1-based vaccine strategies has shown promise in eliciting immune responses in preclinical trials, leading to clinical trials in several cancers, including PDA, that have demonstrated a range of effects on humoral and T cell responses and patient survival (Gao et al., 2020).

Aberrant glycosylation and splicing of mucins can result in longer glycan chains, physically hindering immune interaction with cancer cells and supporting tumor progression and immunosuppression by shielding cancer-associated epitopes (Fig. 3; Jahan et al., 2018). MUC4 provides steric hindrance via its bulky, glycosylated extracellular region to mask immune cell surface antigens and shield tumor cells from immune recognition, thus enhancing survival and extravasation of metastasized tumor cells via interaction with macrophages and hematopoietic progenitors (Rowson-Hodel et al., 2018). Truncated mucin-type *O*-glycans are observed in many epithelial cancer cells and premalignant lesions in adenocarcinomas (Itzkowitz et al., 1991; Ching et al., 1993; Lyubsky et al., 1988; Kim et al., 2002; Tarp and Clausen, 2008; Stanley, 2011). In an immunological context, the truncated glycans Tn and sTn prevent *O*-linked glycan elongation beyond the initial GalNAc residue, increasing sensitivity to NK cell–mediated antibody-dependent cellular cytotoxicity (ADCC) and cytotoxic T-lymphocyte–mediated killing (Madsen et al., 2013). Understanding how aberrant glycosylation contributes to the immunosuppressive microenvironment of PDA will be critical in the design of therapeutic strategies targeting these alterations.

### Leveraging glyco-epitopes in cancer immunotherapy

Recent years have demonstrated effective reactivation of T cell immunity via blockade of the immune checkpoint PD-1/PD-L1 pathway, thereby improving survival in patients with various types of cancer (Chen and Han, 2015; Sharma and Allison, 2015; Topalian et al., 2016; Qin et al., 2019). Unfortunately, many patients only partially respond to PD-1/PD-L1 inhibition (Zou et al., 2016; Topalian et al., 2015). It is critical to understand how post-translational modifications, such as *N*-linked glycosylation, regulate PD-L1. PD-L1 is *N*-glycosylated in melanoma as well as breast, lung, and colon cancer (Li et al., 2016). *N*-linked glycosylation of PD-L1 enhances its protein stability and plays a vital role in PD-1/PD-L1–mediated tumor immunosuppressive function (Li et al., 2016; Hsu et al., 2018; Wang et al., 2018; Cha et al., 2019). Glycosylated PD-L1 suppresses T cell activity, whereas non-glycosylated PD-L1 exhibits less immunosuppressive activity, resulting in slower tumor growth (Li et al., 2018). It will be critical to extend these studies to PDA to understand if altered PD-L1 glycosylation contributes to immunosuppression and poor immune checkpoint blockade therapy response rates.

Current strides in immunotherapies and anti-tumoral immunity involve utilizing glycosite-targeting antibodies to block immunosuppressive interactions and glycoepitope-induced dendritic cell responses to boost T lymphocyte activation in PDA and other cancers. To determine how *O*-glycosylation impacts steric hindrance by mucins, the binding of an anti-MUC1 monoclonal antibody HMFG-2 was assessed in breast, colon, and pancreatic cancer cell lines. HMFG-2 reactivity against MUC1 increased following inhibition of *O*-glycosylation using benzyl-α-*N*-acetylgalactosamide to prevent glycan chain elongation. De-sialylation of cell surfaces also improved access to MUC1 (Ho et al., 1995). The immunodominant DTR (Aspartic Acid-Threonine-Arginine) motif of MUC1, which comprises part of the exposed core peptide resulting from aberrantly truncated glycan chains on MUC1, has been the primary target of many immunotherapeutic interventions, and prior work had uncovered enhanced antibody binding to peptides *O*-glycosylated with GalNAc in the DTR motif (Thr-10) compared to non-glycosylated peptides (Karsten et al., 1998; Karsten et al., 2004). Pankomab, an antibody targeting this carbohydrate-induced conformational tumor-associated epitope on MUC1, features the highest glycosylation dependency and strongest additive binding effect compared to past MUC1 antibodies (Danielczyk et al., 2006). Upon binding, it induces an elevated cytotoxic T-lymphocyte response on tumor cells, and MUC1–chimeric antigen receptor T cells show target-specific cytotoxicity and tumor inhibition in pancreatic cancer xenograft models (Cai et al., 2015).

Immunotherapeutic strategies that target other glycoproteins are also showing promise. CD24, a cell surface sialoglycoprotein expressed by most B lymphocytes, is overexpressed in many human carcinomas. Prior studies have shown that CD24-specific monoclonal SWA11 therapy effectively impeded lung and pancreatic tumor growth in xenotransplanted mice (Bretz et al., 2012). Furthermore, SWA11 therapy not only decreased lung and ovarian carcinoma tumor cell proliferation and affected the tumor microenvironment cytokine milieu in mice, but also results in increased tumor infiltration of macrophages (Salnikov et al., 2013). The cumulative tumor-promoting and restraining effects of CD24 targeting by monoclonal therapy will be critical to explore in autochthonous mouse models of cancer in the context of standard chemotherapeutic intervention.

Other novel approaches to cancer immunotherapy involve postsurgical vaccination with tumor antigen-loaded dendritic cells (Palucka and Banchereau, 2013; Dodson et al., 2011). Fucose-rich glycovariants of bile salt–dependent lipase (BSDL) are expressed during pancreatic tumorigenesis (pBSDL-J28; Mas et al., 1997). Treating mice with dendritic cells loaded with pBSDL-J28 induced T-lymphocyte activation, prevented Panc02 tumor development, and provided long-term protection against Panc02 tumor formation (Collignon et al., 2015). Overall, glycosylation alterations have enormous potential in immunotherapy, but further exploration is necessary to understand how to leverage these targets for the treatment of PDA.

## Conclusions looking forward

Immense strides in glycobiology research have expanded our understanding of glycosylation in cancer biology and have cemented aberrant glycosylation as a key feature in tumor onset and progression. Associations between the presence of aberrant glycan moieties and malignant disease progression are well-documented, with some demonstrating clinical relevance serving as biomarkers of disease progression. Given the dynamic and complex mosaic of glycans and glycoconjugates and their multifaceted regulation, continuing research is required to further understand their functional and causative roles during discrete stages of disease progression. This understanding will reveal vulnerabilities in proliferative signaling, metastatic capabilities, and immune escape that can serve as promising combinatorial therapeutic targets. The emergence of model systems that faithfully recapitulate the human glycome and the heterogeneity observed across patients, such as mouse models of pancreatic disease that produce CA19-9, tissue slices, and human patient-derived organoids, will be crucial in this effort and may reveal critical avenues for therapeutic intervention.
